# Rapid Electrochemical Flow Analysis of Urinary Creatinine on Paper: Unleashing the Potential of Two-Electrode Detection

**DOI:** 10.1021/acssensors.3c01640

**Published:** 2023-09-27

**Authors:** Léonard Bezinge, Niklas Tappauf, Daniel A. Richards, Chih-Jen Shih, Andrew J. deMello

**Affiliations:** Institute for Chemical and Bioengineering, Department of Chemistry and Applied Biosciences, ETH Zürich, Vladimir-Prelog-Weg 1, 8093 Zürich, Switzerland

**Keywords:** flow injection analysis, paper-based microfluidics, laser-induced graphene, electrochemical biosensor, urinary creatinine

## Abstract

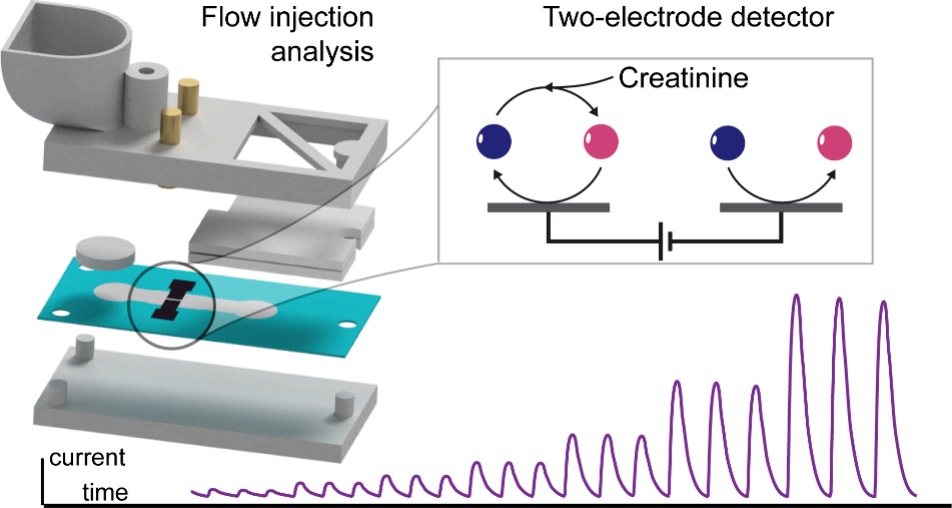

The development of low-cost, disposable electrochemical sensors is an essential step in moving traditionally inaccessible quantitative diagnostic assays toward the point of need. However, a major remaining limitation of current technologies is the reliance on standardized reference electrode materials. Integrating these reference electrodes considerably restricts the choice of the electrode substrate and drastically increases the fabrication costs. Herein, we demonstrate that adoption of two-electrode detection systems can circumvent these limitations and allow for the development of low-cost, paper-based devices. We showcase the power of this approach by developing a continuous flow assay for urinary creatinine enabled by an embedded graphenic two-electrode detector. The detection system not only simplifies sensor fabrication and readout hardware but also provides a robust sensing performance with high detection efficiencies. In addition to enabling high-throughput analysis of clinical urine samples, our two-electrode sensors provide unprecedented insights into the fundamental mechanism of the ferricyanide-mediated creatinine reaction. Finally, we developed a simplified circuitry to drive the detector. This forms the basis of a smart reader that guides the user through the measurement process. This study showcases the potential of affordable capillary-driven cartridges for clinical analysis within primary care settings.

Electrochemical biosensors have the potential to transform biomarker analysis by replacing bulky laboratory equipment with portable devices.^1^ This transition would allow routine clinical tests to be performed in primary care settings, facilitating early disease detection and timely intervention. Over the past decade, activities have shifted toward fully integrated microsystems, with increasing research efforts dedicated to developing low-cost, disposable devices made from accessible materials, such as plastic or paper.^2^ Unfortunately, the fabrication of the reference electrode in these low-cost electrochemical cells remains a significant and unmet challenge.^3^ A typical electrochemical detector includes a reference electrode that acts as a reference potential for the working electrode.^4^ To ensure measurement stability, the reference electrode is usually made by a standard material with a known redox potential, such as silver/silver chloride or the saturated calomel electrode.^4^ However, these materials are often incompatible with common microfabrication techniques or demand complex fabrication processes, thus hindering their integration into low-cost disposable cartridges.^3,5^

Circumventing the overreliance on reference electrodes would enable researchers to design electrochemical sensors using a far broader array of substrates and thus pave the way toward simpler and more accessible diagnostic technologies. A popular approach is to use an uncoated electrode material, such as carbon or gold, as the reference electrode (i.e., pseudoreference electrodes).^6−9^ However, because the electric potential of pseudoreference electrodes strongly depends on their chemical environment, this approach fails to deliver a consistent reference potential in continuous flow detection assays.^5^ As a result, the integration of dedicated silver/silver chloride electrodes remains the norm in-flow injection analysis (FIA), hindering the fabrication of low-cost disposable devices.^10^

FIA is a widely used analytical technique that enables reliable and high-throughput testing of multiple samples by injecting them into a continuous flow; its rapid and automated nature makes it particularly attractive for clinical chemistry.^11^ In the realm of point-of-need testing, paper-based FIA devices have attracted considerable attention due to low fabrication costs and their passive, pump-free operation.^2,12^ Because of the difficulty of fabricating electrodes in cellulose paper, early attempts to incorporate electrochemical sensing into paper-based FIA relied on the use of external electrodes in contact with the fluidic path.^12−14^ Such a strategy essentially undermines the advantage of paper as a single-use substrate^15^ since the external electrodes are reused multiple times, raising the risk of cross-contamination. To address this shortcoming, we recently developed laser-pyrolysis of cellulose as a versatile technology for fabricating electrodes fully embedded into paper.^16^ Despite their advantages, the electrochemical sensors fabricated in this manner still relied on a three-electrode configuration, with a laser-induced graphenized (LIG) pseudoreference, resulting in signal aberration in continuous flow assays.^16^

The adoption of two-electrode, or reference-free, electrochemical sensors has the potential to address many of the challenges associated with the use of pseudoreferences in FIA. Moving to two-electrode systems would substantially simplify device fabrication and readout hardware by eliminating the need for a reference potential altogether.^5,17^ Unfortunately, due to their unconventional nature and our limited insights into their workings, two-electrode systems remain underexplored and are often used empirically without rational design.^18,19^ Accordingly, understanding the inherent limitations of these systems is essential to harnessing their full potential and ensuring their widespread adoption. Here, using paper-based laser-pyrolyzed devices as a model system, we demonstrate that two-electrode detection can provide excellent performance in FIA cartridges for clinical analysis. As a proof of concept, we designed a cartridge to perform urinary creatinine analysis, an essential diagnostic assay^20^ in need of miniaturization and automation.^21^ Urinary creatinine serves as a key biomarker for kidney function as its production is steady and proportional to a healthy individual’s muscle mass.^22,23^ It is also widely used as a normalizing factor in urine assays, such as the albumin-to-creatinine ratio, or as an internal control for sample collection adequacy.^24^ The gold-standard method for quantifying urine creatinine remains the Jaffe reaction, which is a colorimetric reaction between creatinine and picric acid developed in 1886 (Figure 1a).^25,26^ However, this assay has limitations, e.g., explosiveness of dried picric acid or cross-reaction with glucose and is not suitable for miniaturization and high-throughput analysis due to the need of bulky and expensive optics for signal readout.^27^ In addition, absorbance-based readout loses quantification as the optical path length decreases in miniaturized systems.^28^ A number of electrochemical approaches for quantitative creatinine analysis have been proposed,^29^ including those based on iron ion mediators,^30−32^ picric acid detection,^27,33^ or direct detection catalyzed by copper nanoparticles.^34^ Unfortunately, these approaches suffer from slow electron transfer, large redox potential, or demanding tedious electrode modification, respectively. Thus, they are unsuitable for use in low-cost disposable devices.

**Figure 1:**
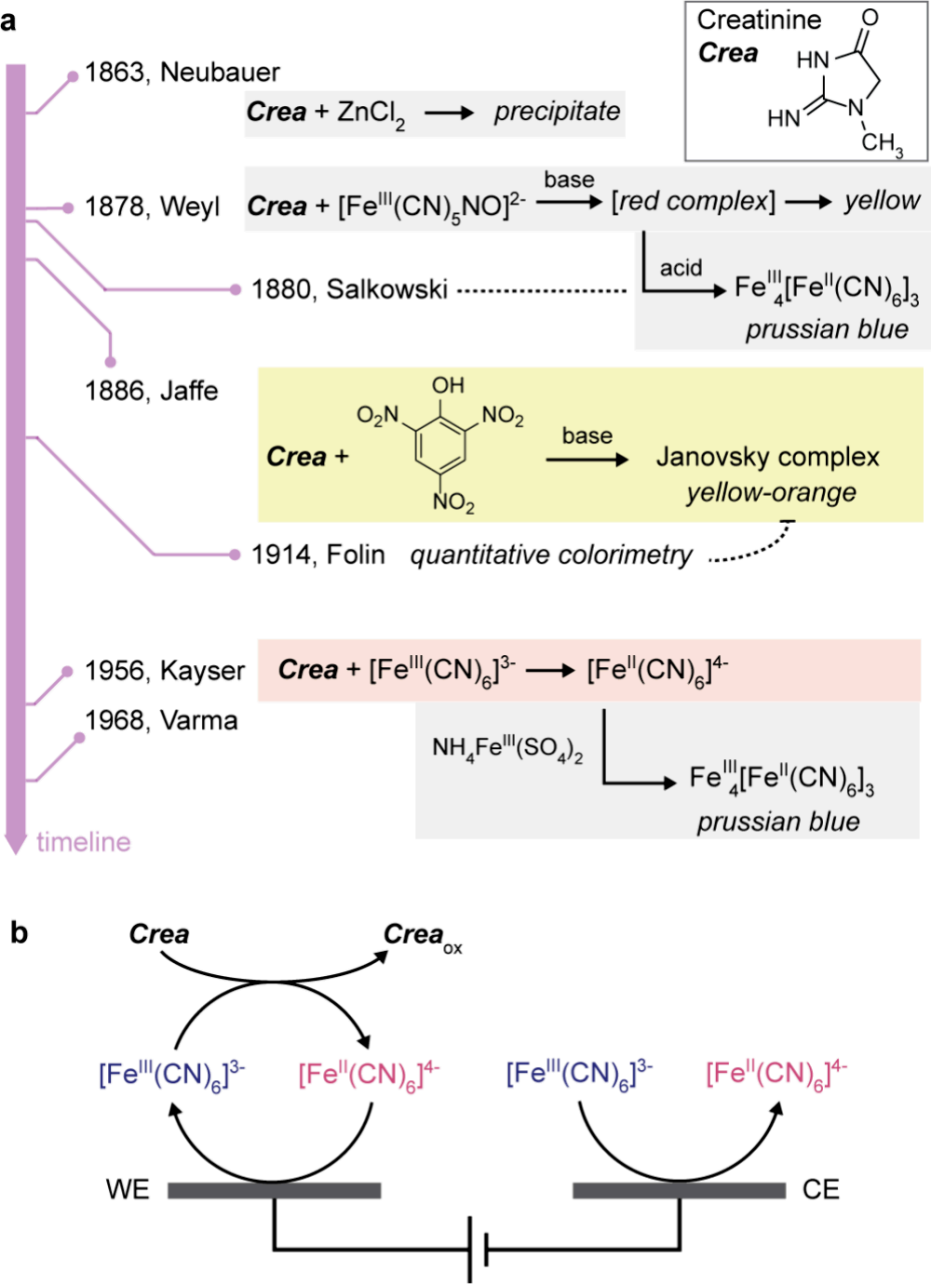
Learning from the past. (a) Historical perspective of colorimetric methods for the detection of urinary creatinine. (b) Schematic diagram illustrating the electrochemical detection of creatinine mediated by ferricyanide on our two-electrode sensors comprising working and counter electrodes, WE and CE.

When searching for a more suitable detection system, we discovered that reactions between ferricyanide complexes and creatinine, which were discovered in the 19th century (Figure 1a),^35−39^ remained relatively unexplored within electrochemical systems despite the attractive features of ferricyanide (fast, reversible, and wide electrode compatibility) for low-cost systems (Figure 1b).^4^ Accordingly, we investigated the ferricyanide-mediated electrochemical detection of urinary creatinine and its implementation in a paper-based FIA with two-electrode detectors. We first explored the working range and limitations of the two-electrode detectors and then optimized their sensing performance for urinary creatinine. Finally, we demonstrated high-throughput testing of clinical urine samples using disposable cartridges and a low-cost reader.

## Results and Discussion

**Figure 2:**
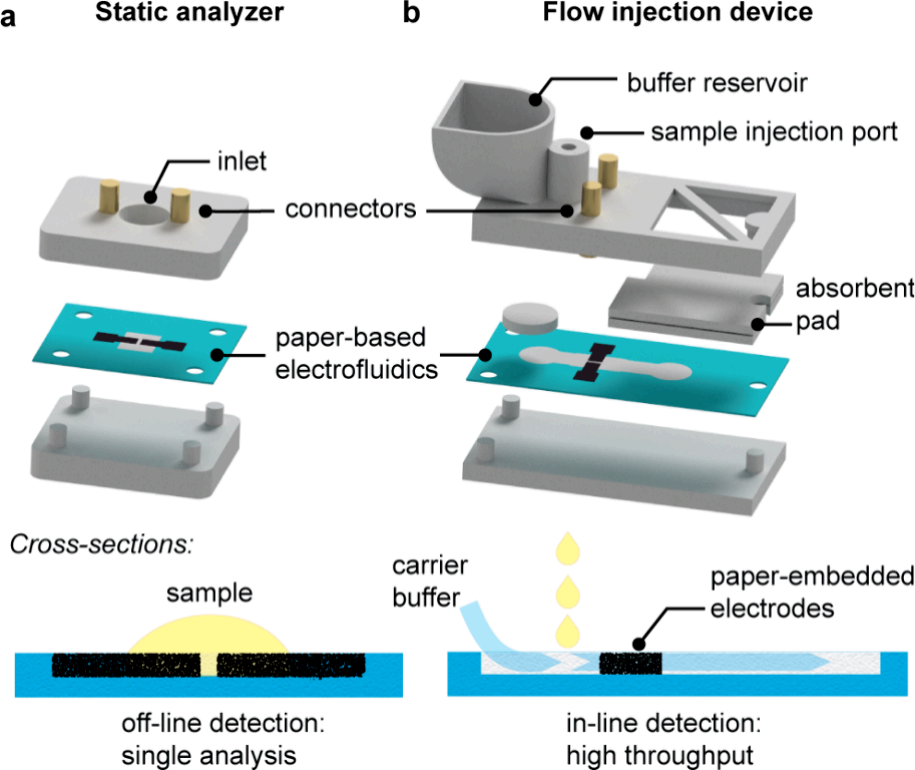
Schematics of the paper-based electrochemical devices developed in this study. (a) Static analyzer is used to examine fundamental properties of two-electrode detection, where the sample is directly drop-cast onto the embedded two-electrode detector. (b) Flow injection device allows for the sequential injection of multiple samples into a continuous stream of carrier buffer solution.

To develop our FIA assays, we utilized two different device designs. Both comprise a paper-based electrofluidic layer with an embedded graphenic two-electrode detector made up of working and counter electrodes (WE and CE) (Figure 2). The simplest device, henceforth referred to as the “static analyzer”, allows for a single measurement of one sample introduced through a wide pipetting window (Figure 2a). We used this device to examine the fundamental properties of two-electrode detection systems and to optimize the electrochemical detection of the ferricyanide-mediated reaction with creatinine. We additionally designed a flow injection device for analyzing multiple clinical samples in a continuous flow format. This device relies on a flow of carrier buffer from a reservoir through the electrofluidic channel before reaching a large-capacity absorbent pad (Figure 2b). In this configuration, samples can be sequentially injected upstream of the detector, offering high throughput and continuous detection of multiple samples.

### Understanding the Limitations of the Two-Electrode Detection

**Figure 3:**
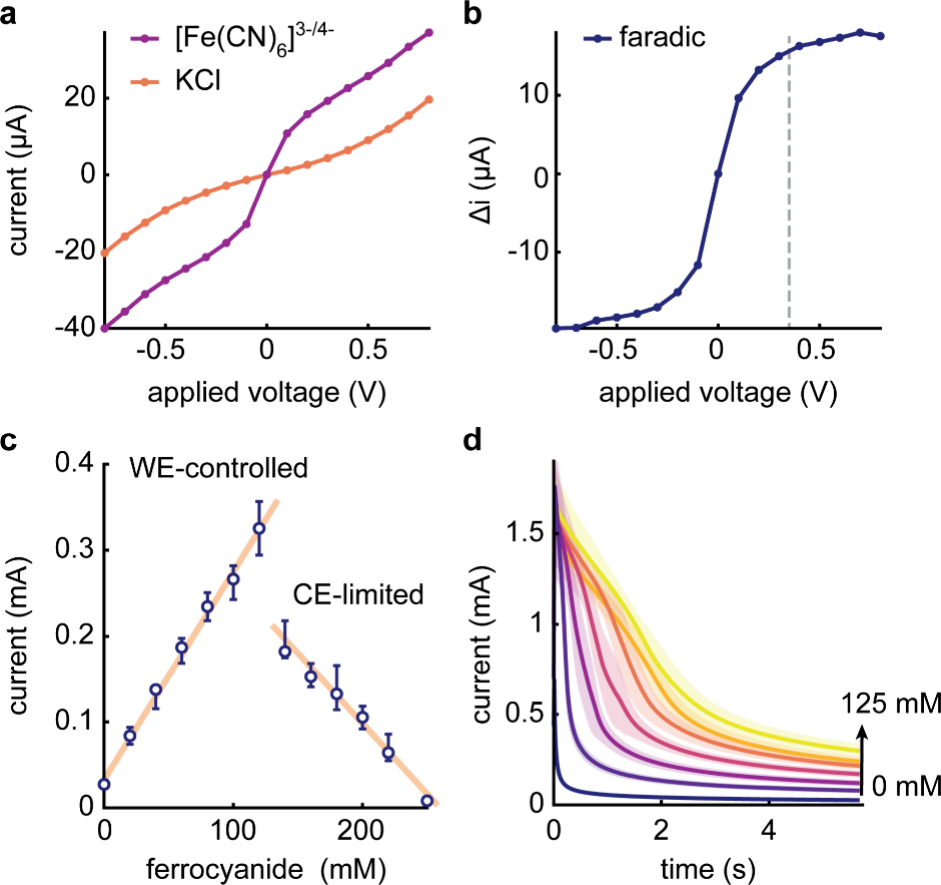
Two-electrode electrochemical detection of ferro-/ferricyanide probes. (a) Representative responses of sampled current as a function of applied voltage in the presence or absence of 10 mM ferro-/ferricyanide (1:1 ratio) in 1 M KCl, using a sampling time of 5 s and a base voltage of 0 V. (b) Faradaic contribution extracted from the measurement in (a). The dashed line at 0.35 V represents the voltage used in all our subsequent chronoamperometric measurements. (c) Calibration curve (*n* = 3) of ferrocyanide for a constant total concentration of 250 mM ferro-/ferricyanide. (d) Chronoamperometric curves (*n* = 3) for 0 to 125 mM ferrocyanide (in the WE-controlled regime).

The theory and design principles for electrochemical sensors have been primarily developed for three-electrode configurations and thus were not entirely applicable to our proposed system, Thus, before focusing on our target analytes, we aimed to gain some insights into two-electrode electrochemical detection, including its working range and limitations. To this end, we first evaluated the optimal voltage for the detection of ferrocyanide by measuring sampled current voltammograms in the presence or absence of redox probes (Figure 3a). This allowed us to extract the faradic current-overpotential response by subtracting nonfaradic contributions (Figure 3b). The response resembles that of a characteristic Nernstian system, revealing fast and reversible electron transfer kinetics and diffusion control.^4^ In subsequent measurements, we chose an applied voltage of 0.35 V, corresponding to approximately 90% of the maximum faradic current attainable, with a small degree of nonfaradic contribution (∼25%). Compared to a typical three-electrode format, the applied voltage in the two-electrode system is larger because it represents the voltage difference between the WE and CE rather than that between the WE and the reference electrode (Figure S1).

It can be seen that when the ferrocyanide ([Fe^II^(CN)_6_]^4–^) concentration varies relative to ferricyanide ([Fe^III^(CN)_6_]^3–^), while maintaining a total concentration of 250 mM, two distinct regimes in the current responses exist (Figure 3c). Up to a ferrocyanide concentration of 125 mM, the oxidation signal is linearly proportional to its concentration and consistent with the Cottrell equation for a conventional cell (*i* = *nFAC*_0_*D*^0.5^*t*^–0.5^π^–0.5^, where *i* and *t* are current and time, respectively, *n* the number of electrons transferred, *A* the surface area of the WE, and *C*_0_ the analyte concentration with diffusion coefficient *D*).^4^ Beyond 125 mM, a sudden current drop is observed, owing to the deficiency of ferricyanide available at the CE for reduction, and thus unable to absorb the electrons generated at the anode. That said, if we consider the complete chronoamperometric curves (Figures 3d, S2, and S3), responses appear to deviate from the classical Cottrell equation, namely, *i* ∝ *t*^–0.5^.^4^ Indeed, a noticeable delay exists in response to high ferrocyanide concentrations. Further modeling efforts are required to fully explain this observation. These results demonstrate the importance of considering the linear regime of two-electrode detectors, as it will ultimately determine the upper detection limit for a given application.

### Unraveling the Mechanism of Ferricyanide-Mediated Creatinine Detection

Despite a long history, the reaction of creatinine with ferricyanide remains relatively unexplored, and mechanistic studies and parametric optimization are lacking.^35−39^ This knowledge gap exists because the reaction is difficult to incorporate into colorimetric analysis since ferrocyanide requires further reaction steps to generate a measurable color change, e.g., with Fe^3+^ to form Prussian blue (Figure 1). By removing this second step, the two-electrode detector format presented here offers an attractive platform for studying the kinetics and mechanisms of this reaction.

**Figure 4:**
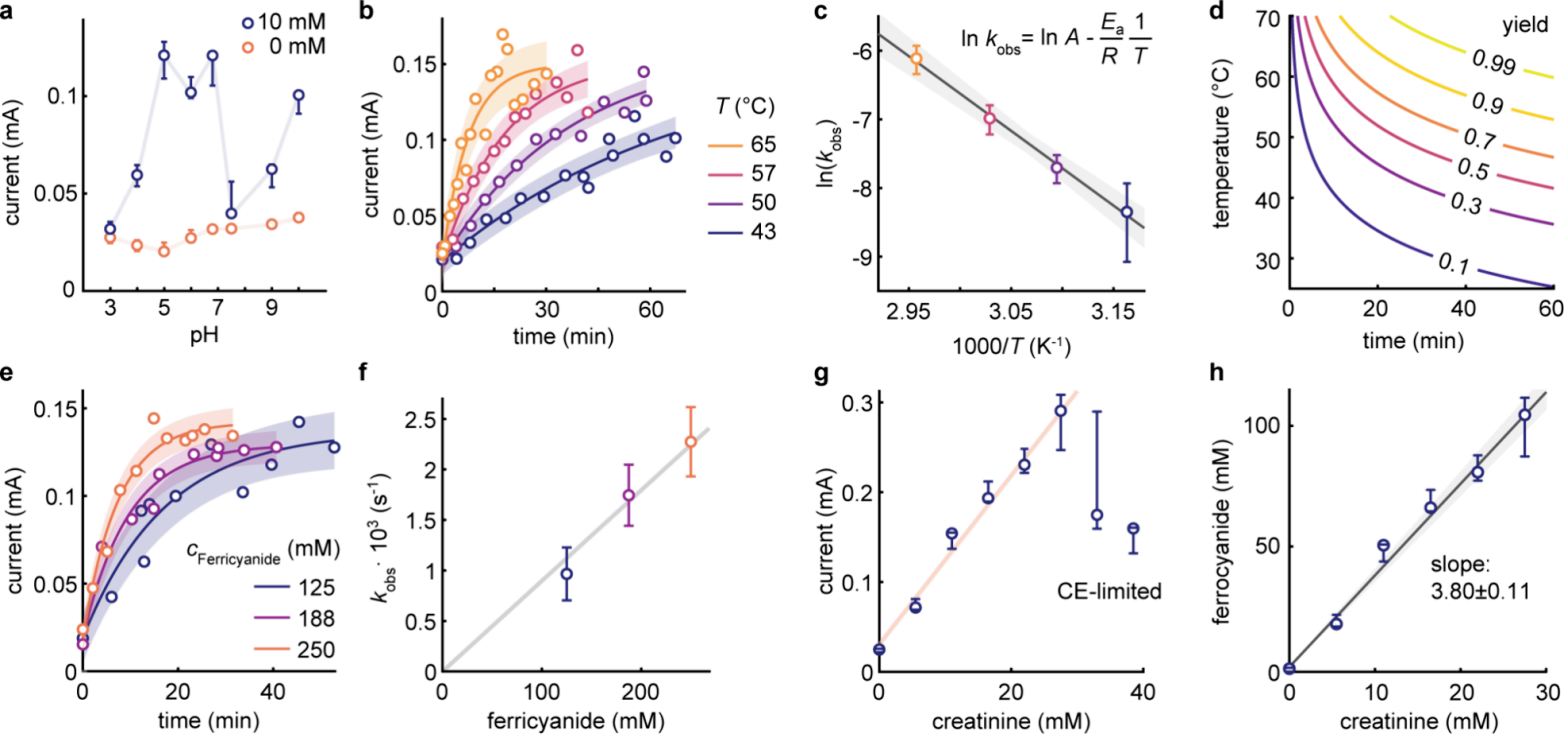
Mechanistic analysis of the ferricyanide-mediated detection of creatinine. (a) Oxidation current measured after 10 min of reaction between 10 mM creatinine with 250 mM ferricyanide in 1 M KCl and at various pH values (*n* = 3). (b) Reaction kinetic responses fitted with *i* = *i*_max_ (1 – exp(−*k*_obs_·*t*)) at pH 5 and various temperatures. (c) Arrhenius plot for the observed reaction rate constants assuming pseudo-first-order kinetics. (d) Calculated reaction yield map as functions of temperature and time for creatinine conversion in 250 mM ferricyanide, 250 mM acetate, pH 5, and 1 M KCl. (e) Reaction kinetic responses for three ferricyanide concentrations. (f) Extracted *k*_obs_ values as a function of ferricyanide concentration. (g) Current signals (*n* = 3) as a function of creatinine concentrations. At high concentrations, response linearity breaks down due to the limitations of the CE. (h) Correlation of sampled current values with ferrocyanide oxidation signals reveals that on average 3.80 ± 0.11 ferricyanide molecules reacted with one creatinine molecule.

Using the static analyzer, we investigated the influence of pH, temperature, and reactant concentrations on the yield and reaction kinetics by measuring variations in oxidation currents. Our analysis revealed that slightly acidic or basic conditions are desirable, with the optimal value at pH = 5 (Figures 4a and S4). Extreme pH values resulted in the undesired formation of Prussian blue (Figure S5). At pH = 5, we fitted the current responses using *i* = *i*_max_ (1 – exp(−*k*_obs_·*t*)), where the fitting parameters of *i*_max_ and *k*_obs_ correspond to the maximum current and observed reaction rate constant, respectively, and found that the fitted *k*_obs_ values are nearly independent of creatinine concentration, indicating pseudo-first-order kinetics (Figure S6).^40^ The observed reaction rates increased with temperature following the Arrhenius equation (Figures 4b,c and S7), with an activation energy, *E*_a_, of 10.9 ± 0.4 kJ mol^–1^ and a pre-exponential parameter, *A*, of 26.0 ± 1.4 s^–1^. Using these values, we created a reaction yield map as a function of time and temperature (Figure 4d). For example, at 65 °C, one can reach a reaction yield of >95% after 25 min.

We then carried out a mechanistic analysis of the reaction, starting with an assessment of the role of ferricyanide in the rate-limiting step (Figures 4e and S8). The observed reaction rates were linearly proportional to the initial ferricyanide concentration (the first-order contribution), indicating that the rate-limiting step involves a bimolecular reaction between creatinine and an excess of ferricyanide (Figure 4f).^40^ We also investigated the impact of creatinine concentration (Figure 4g). The response reveals a linear dependence up to 27.5 mM, beyond which the current suddenly drops due to CE limitations. By correlating the sampled current values in the linear region with the ferrocyanide oxidation signals calibrated in Figure 3c, we estimate that, on average, 3.80 ± 0.11 ferricyanide molecules reacted with one creatinine molecule (Figure 4h). In other words, the total reaction corresponds to the oxidation of two chemical bonds per creatinine molecule, leading to the reduction of four ferricyanide molecules. In light of these findings, we hypothesize that the kinetics involve four sequential steps, in which one creatinine molecule is progressively oxidized by one ferricyanide molecule at each step. The analysis presented here not only identifies the optimal reaction conditions but also offers a rationale for the system limitation. More specifically, one must keep the ferricyanide concentration at least eight times (2 × 4) higher than the maximum creatinine concentration to be detected, taking into account the dilution factors when implemented in a sample matrix (such as urine).

### Performance Optimization through Electrode Design

Electrode design plays a critical role in determining the electrochemical performance since signals are generally proportional to the electroactive area and the electrode arrangement can enhance signal quality by mitigating diffusion and migration pathways.^4^ This is particularly true for two-electrode detection schemes, as both the CE and WE design will affect overall detection efficiency, and our results suggest that there is a subtle balance between signal strength and sufficient counter reaction.

**Figure 5:**
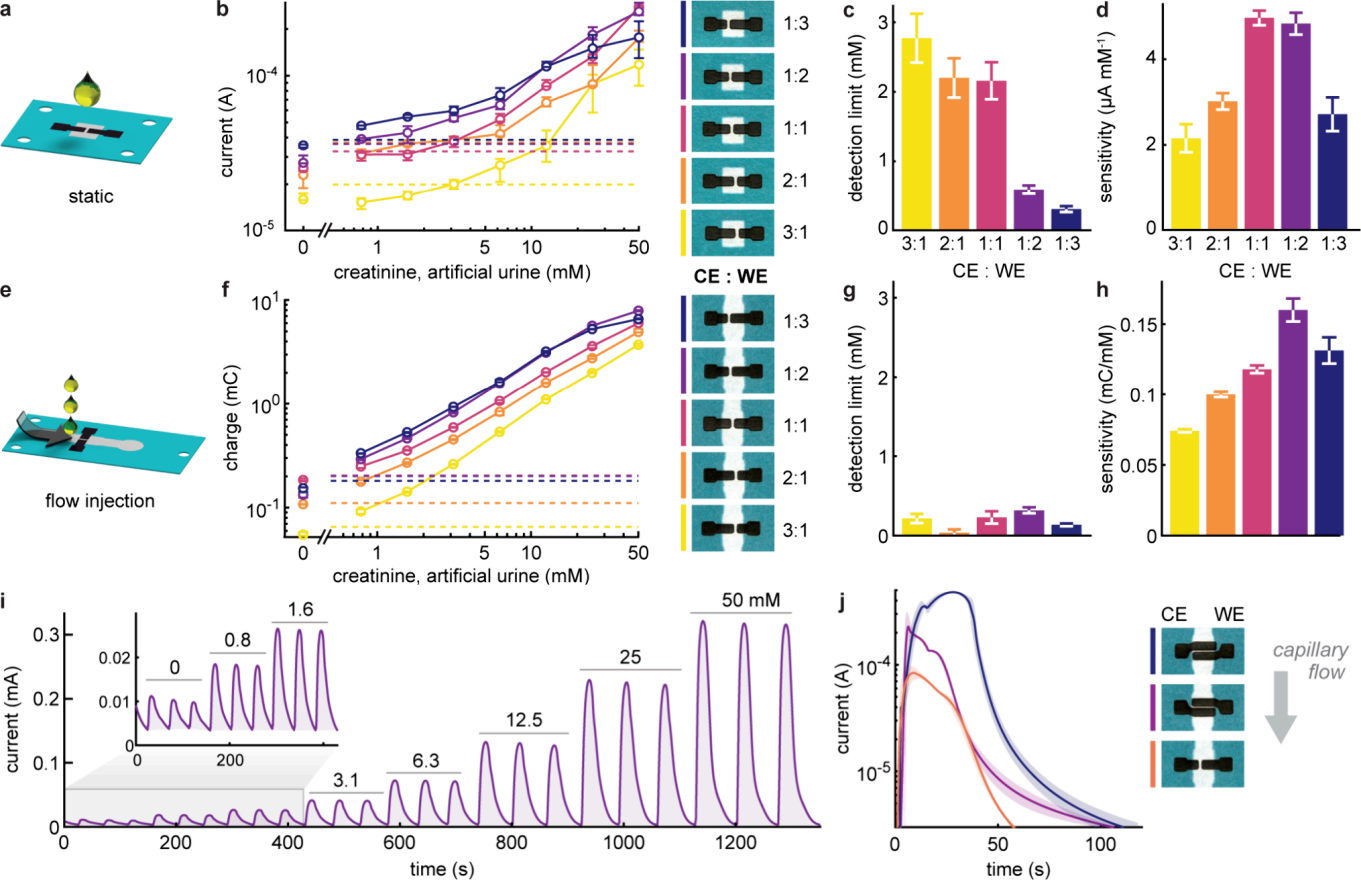
Optimizing electrode design for sensing performance. Artificial urine samples spiked with creatinine were analyzed using the static analyzer (a–d) and the flow injection device (e–j), with an effective dilution factor of 2 after addition of buffer and redox mediator. (a) Schematic diagram for sampling in a static analyzer. (b) Characterized current signals (*n* = 3) from the chronoamperometric measurements at 0.35 V as a function of creatinine concentration for various CE:WE area ratios. The dashed lines correspond to the respective current thresholds determined by the negative-control measurements plus three standard deviations. The detection limits (c) and calibration sensitivities (d) for individual electrode designs were derived from the response curves. (e) Schematic diagram for sampling in a flow injection device. (f–h) Similar experiments were carried out with a constant applied voltage of 0.35 V. For each creatinine concentration, samples were injected in triplicate. (i) Representative current–time response for a complete measurement sequence at the 1:1 CE:WE ratio. The current–time integrals (shaded areas) correspond to the signals. (j) Comparison of the signal responses for three-electrode arrangements following the injection of an artificial urine sample containing 6.3 mM creatinine (*n* = 3). Photographs of all electrode designs are shown in (b), (f), and (j). In each device, the channel width is 4 mm.

Using the static analyzer, we evaluated sensor performance on artificial urine samples spiked with creatinine, while varying the CE:WE area ratio (Figures 5a,b and S9).^41^ Interestingly, although increasing the relative WE area amplifies the signal and lowers the detection limit (Figure 5c), response linearity and analytical sensitivity are gradually lost due to the insufficient CE size (Figure 5d).^42^ We found that a CE:WE of 1:2 showed optimal performance, with a detection limit of 0.56 ± 0.07 mM and an analytical sensitivity of 4.83 ± 0.25 μA mM^–1^.^42^ Note that in addition to magnitude, the standard deviation of the sensitivity values indicates the response linearity of the system.^42^

When compared to the static analyzers, paper-based flow injection analyzers offer a number of key advantages (Figures 5e,f and S10). First, they allow the injection of multiple samples into a single device (at rates in excess of 1 sample per minute), substantially reducing reagent consumption (carrier flow rate of 7–12 μL min^–1^, Figure S11) and sample volumes (down to 1 μL, Figure S12). Second, the device-to-device variation is eliminated since all measurements can be performed on a single device, leaving only variations resulting from the injection process, which is approximately 6% in our FIA devices (Figures S13 and S14). Additionally, the signals (unit: Coulomb) are quantified by integrating currents over time as the sample flows through the electrode, thus averaging the noise from the measurement readout. Surprisingly, and despite the weaker signals generated by the smaller WEs, we observed detection limits below 0.32 mM, regardless of the relative electrode area (Figure 5g). This is due to the ability of the FIA device to detect small deviations from the steady baseline established by the carrier flow. On the other hand, the sensitivity shares a similar trend with that of static analyzers, showing a reduced sensitivity with decreasing CE area (Figure 5h).

Figure 5i presents a complete measurement sequence in FIA using a 1:1 CE/WE electrode size ratio. The continuous flow sensor delivers a detection limit of 0.23 ± 0.08 mM and sensitivity of 0.118 ± 0.003 mC mM^–1^ up to 50 mM creatinine, with the concentration dependence showing only a 2.5% deviation from perfect linearity. We attribute the remarkable FIA performance to the high efficiency of in-flow detection with integrated graphenic electrodes. More specifically, the porous and embedded nature of our laser-pyrolyzed electrodes, together with the fast kinetics of ferrocyanide at the interface (*k*_0_ = 0.011 ± 0.003 cm s^–1^),^16^ result in overall detection efficiencies of up to 56% (Figure S15). If we further account for the partial coverage of the WE in the channel, the observed efficiencies reached up to 90% of the maximum attainable values, meaning that up to 90% of the redox probes flowing through the WE were successfully detected.

It is also noteworthy that when two electrodes are placed in a parallel arrangement, the response time is significantly shorter than that for sequential designs, which results in undesirable redox cycling (Figures 5j and S16). In summary, we have taken advantage of the rapid-prototyping capability offered by the laser-pyrolyzed electrodes to investigate and optimize electrode design in our devices. Our findings reveal that the electrode areas and orientation directly affect the response in two-electrode detection. In FIA, the two-electrode detectors not only enable high-throughput sampling but also showcase improved sensing performance and high detection efficiencies.

### High-Throughput Flow Injection Analysis of Clinical Samples

**Figure 6:**
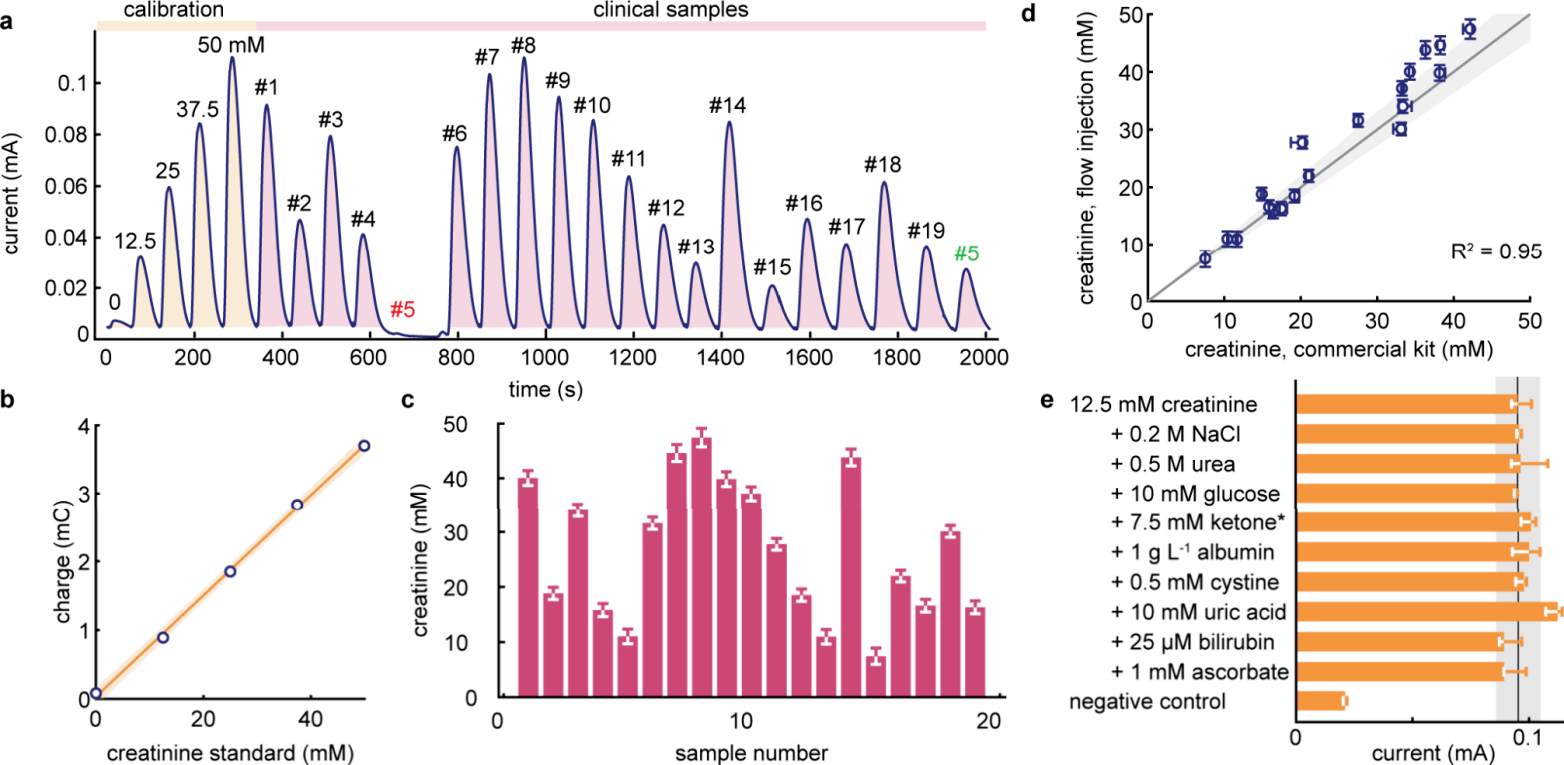
High-throughput analysis of clinical urine samples. (a) Nineteen urine samples collected from feline or canine specimens in veterinary settings were sequentially analyzed with our flow injection device (WE:CE of 1:1). Analysis starts with the injection of five calibration standards (creatinine in water), followed by the clinical urine samples. The shaded surface areas correspond to the peak integrals. Sample #5 was pinned to the injection port due to its higher viscosity and reinjected after the last sample. Using the calibration curve (b), the creatinine content in the clinical samples could be determined (c). (d) Creatinine content values determined by our device are in excellent agreement with those obtained by the gold-standard plate-based method, with an average absolute deviation of 11%. The shaded gray area represents ±10% deviation from perfect agreement. (e) Interference analysis for a selected panel of substances spiked into the artificial urine samples containing 12.5 mM creatinine, measured on our static devices (*n* = 3). The gray area represents ±10% deviation from the positive control (ketone*: 3-hydroxybutyrate).

A major advantage of FIA in testing clinical samples with unknown creatinine content is that the device does not require prior calibration. Instead, it incorporates internal calibration and control measurements that effectively remove detrimental analytical effects caused by device-to-device or reagent variations. To examine the reliability and accuracy of our two-electrode FIA devices, we analyzed 19 clinical urine samples collected in veterinary settings to determine their creatinine content and benchmarked our results against a commercial colorimetric assay (Table S1, Figure 6d). A typical set of measurements starts with the injection of five standard creatinine solutions with known concentrations followed by the urine samples on the same device (Figure 6a).

Using the internal standards, we directly determined the creatinine content in each clinical sample (Figure 6b,c) which ranged from 7 to 47 mM, covering the entire clinical (high to low) range.^22,24^ Comparing our results to those benchmarked with the gold-standard commercial colorimetric kit using a laboratory plate reader (Figure S17), our paper-based two-electrode FIA cartridges exhibit excellent agreement, with an average absolute deviation of +2.2 mM, or 11% (*R*^2^ = 0.95; Figures 6d and S18). In addition, we examined a selected panel of possible interferences at abnormally high concentrations (Table S2) and, apart from uric acid (+18%), found insensitive response for all interfering compounds (<10% signal deviation, Figure 6e).

It is noteworthy that the real-time measurement of our FIA assay can quickly identify abnormal sampling. For example, in Figure 6a, sample #5 was unusually viscous and became pinned to the injection hole. The absence of signal allowed us to quickly identify the failure and resolve it by testing at a later stage without interrupting the analysis workflow. In summary, our low-cost two-electrode cartridge demonstrated accurate, high-throughput testing of urine samples in a clinical scenario.

### Developing a $3 Smart Reader

**Figure 7:**
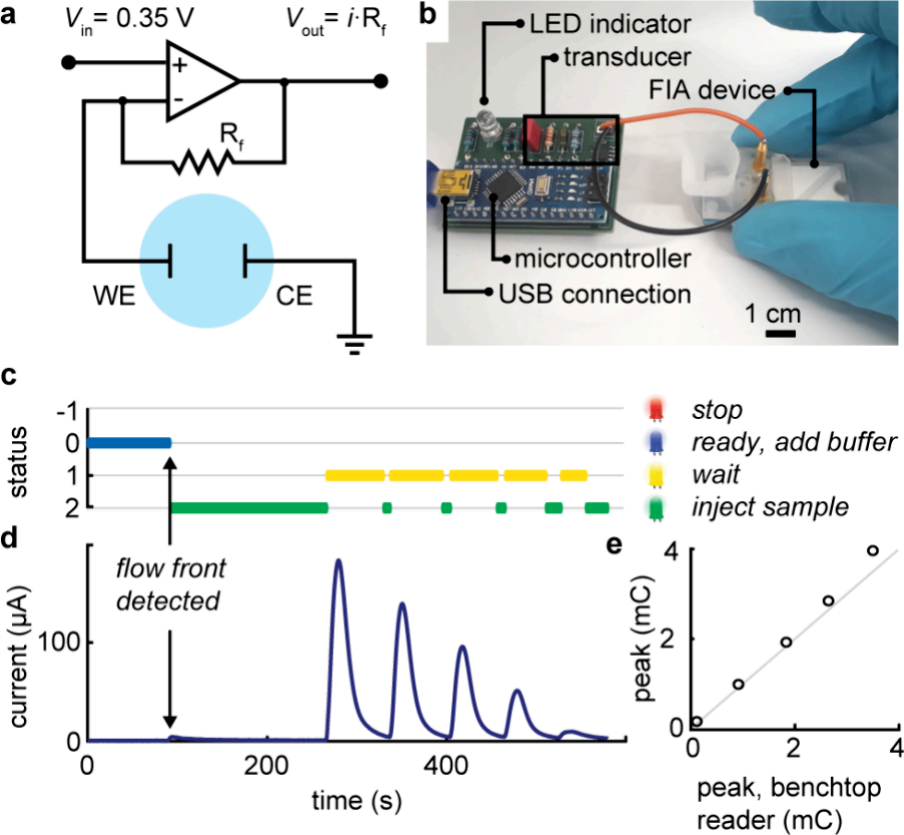
$3 smart reader driving our two-electrode detector. (a) Circuit diagram of the electrochemical transducer. A *V*_in_ potential of 0.35 V is applied to the WE, and the current is monitored via the output voltage (*V*_out_) using an operational amplifier. The current range is tuned by the feedback resistance *R*_f_. (b) Photograph of the smart reader, which includes a microcontroller that carries out real-time measurements and guides the user with a multicolor indicator LED. (c, d) In a typical analysis, the smart device detects the flow front and informs the user of the status of the detection and when to inject the next sample. (e) System delivers results consistent with those using a high-precision benchtop potentiostat.

The development and implementation of our two-electrode systems not only greatly simplify the fabrication process but also leads to simplified electronic circuitry for driving and reading the electrochemical transducer.^4,17^ Indeed, due to the fixed potential of the CE, it is not necessary to variably adapt any potentials relative to a reference electrode, as is the case in three-electrode systems. This means that fewer components are required to create and operate these two-electrode systems. Since open-source three-electrode potentiostats require at least $40 worth of parts,^43^ we see enormous potential for two-electrode systems in significantly lowering the costs associated with creating accessible signal readers. To this end, we designed a reader using only one operational amplifier to apply a constant voltage (361.76 ± 0.05 mV) and monitor the current with 0.2 μA resolution and a range up to 211 μA (Figures 7a, S19, and S20). The current range (with constant 0.1% resolution) can be tuned by swapping a single resistance and tailored to a specific application. The system is controlled by an inexpensive microcontroller, resulting in a total part cost of less than $3 (Table S3). Remarkably, we can harness the computing power of the reader not only to drive the transducer but also to analyze results in real time and guide the user through the measurement process using a multicolor LED (Figure 7b and Supplementary Video 1).

In a typical experiment, the reader remains idle until it detects the flow front from the carrier buffer (Figure 7c,d) because of a change in resistivity. It then proceeds to detect the peaks and notifies the user when the current has sufficiently decayed and the cartridge is ready for the next injection. We also incorporated a stop criterion, which currently simply relies on a time limit but could detect critical system failures in the future. Remarkably, and despite its simplicity, the $3 reader delivers performance comparable with high-precision benchtop potentiostats (Figure 7e). Furthermore, the smart reader streamlines the testing process for operators and offers the flexibility of standalone operation or real-time communication of results to a display, laptop, or smartphone via a USB connection (Figure S21).

## Conclusions

In conclusion, we have demonstrated the remarkable performance of two-electrode electrochemical detectors integrated in low-cost clinical analyzers. The two-electrode detectors are ideally suited for paper-based devices, where the fabrication of reference electrodes is cumbersome. The two-electrode sensors also excel at flow injection analysis, thanks to their high detection efficiency and reliable sensing performance. We have confirmed the critical role of the counter electrode design in order to ensure a sufficient number of redox probes for the reverse reaction. Importantly, the two-electrode system is capable of handling the entire development workflow of a novel assay. As a model system, we successfully developed an FIA assay for urinary creatinine, providing unprecedented insights into the ferricyanide-mediated reaction and offering clinical performances comparable with commercial laboratory assays, at almost zero material cost. The cost competence is even further strengthened with our $3 smart reader owing to the simplified circuitry for driving two-electrode systems. We believe that our findings will be readily translatable to other ferricyanide-mediated assays such as glucose or lactate^44^ and anticipate that the ultralow cost paper-based cartridges presented here will facilitate the development of quantitative assays at the point of need.

## Data Availability

All data are available in the main text or the Supporting Information. The design files for digital fabrication of all devices presented in this study as well as the code for the two-electrode potentiostat, including a Python graphical interface, are available at 10.17632/gzj9jz2cnv.1.

## Supporting Information Available

Experimental methods, cyclic voltammetry characterization, negative control experiments, additional concentrations and temperatures, flow injection analysis study, chronoamperometry measurements, stability and performance analysis, colorimetric clinical sample testing, graphical user interfaces, circuit schematics, clinical testing analysis, selection of interferences, and reader cost analysis (PDF)
Experiment with the smart reader (MP4)
