# Supplementary material for: Rapid Electrochemical Flow Analysis of Urinary Creatinine on Paper: Unleashing the Potential of Two-Electrode Detection
Source: ACS Sens. 2023 Sep 27;8(10):3964–72. doi: 10.1021/acssensors.3c01640 (PMC10616850; doi:10.1021/acssensors.3c01640)
Supplement: Supplementary file 1 — se3c01640_si_001.pdf [file se3c01640_si_001.pdf]

Supporting Information

Rapid electrochemical flow analysis of urinary creatinine on paper: unleashing the potential of two-electrode detection

Léonard Bezinge, Niklas Tappauf, Daniel A. Richards*, Chih-Jen Shih* and Andrew J. deMello*

Institute for Chemical and Bioengineering, Department of Chemistry and Applied Biosciences, ETH Zürich, Vladimir-Prelog-Weg 1, 8093 Zürich, Switzerland.

* andrew.demello@chem.ethz.ch, chih-jen.shih@chem.ethz.ch, daniel.richards@chem.ethz.ch

Content

Experimental Methods S2
- Chemicals S2
- Device fabrication S2
- Electrochemical measurements S2
- Electrochemical bioassay S3
- Clinical samples S3
- Statistical analysis S4

Supplementary Figures S5
Supplementary Tables S17
Supplementary Videos S19
References S19

Experimental Methods

Chemicals

Creatinine (98%) was acquired from AlfaAesar. Potassium ferricyanide(III) (99+%) and Tween 20 were obtained from Acros Organics. Acetic acid (>99.99%), albumin from bovine serum (BSA, >96%), ammonium sulfamate (>98%), potassium ferrocyanide(II) trihydrate (>99%), potassium chloride (>99.5%), potassium acetate (>99%) and were purchased from Sigma Aldrich. All chemicals were used as received without further purification.

Device fabrication

The electrofluidic layer was fabricated as previously reported.[1] In brief, cellulose paper (CF3, Cytiva) was treated with ammonium sulfamate (0.8 M, 92 g L^{-1}) and pyrolyzed using a 100 W CO_2 laser engraver (Speedy 300, Trotec) with power setting 18 (measured 4.8 W), speed 5 (measured 16 cm s^{-1}), 10 mm defocus using a 1.5 inch lens, 1000 laser pulses per inch and 333 lines per inch, under nitrogen atmosphere. The embedded electrodes were rinsed with water, isopropanol and acetone, and dried overnight. The electrode layers were treated with oxygen plasma for 90 seconds at 90 W (Atto, Diener Electronic), and the channels laminated at 110°C from wax patterns deposited on a transparency (Xerox Colorqube 8570). All designs were created in Adobe Illustrator CS6. The device holders were 3D printed (SL1S, Prusa) and spring connectors were inserted and glued into the holders (5110/S-C-1.5N-AU-2.3 C connectors, PTR Hartmann). Thick cellulose pads (CF6, Cytiva) were laser cut to shape and used as absorbent and buffer pads. See Data and materials availability for access to all fabrication design files.

Electrochemical measurements

Unless mentioned otherwise, electrochemical measurements were performed using a potentiostat (PGSTAT202, Metrohm Autolab) in chronoamperometry mode with shorted CE and RE, with an applied voltage of 0.35 V and a sampling resolution of 10 ms. For measurements on the static devices, signals are extracted from the current value after 5 seconds. In flow injection analysis, the signals were computed from the integrated current peaks after linear baseline subtraction. The detection efficiency, η, was computed as $\eta = {n_{\mathrm{EChem}}}/{n_{\mathrm{inj}}}$, where n_{EChem} and n_{inj} represent the number of moles of analytes

that were detected and injected, respectively. These two values were calculated from $n_{\mathrm{EChem}} = (S_{\mathrm{p}} - S_{\mathrm{p}}^{0})/(n \cdot F)$, and $n_{\mathrm{inj}} = (3.80\ C_{cr}\ V_{\mathrm{inj}})/Dil$, with S_{p} and S_{p}^{0} being the integrated peaks (in Coulomb), F the faraday constant, $n = 1$ the number of electrons involved in the reaction, $3.80\ C_{\mathrm{Cr}}$ the concentration of ferrocyanide assuming 3.8 mol generated per mole of creatinine, V_{inj} the injected volume, and *Dil* the dilution factor.

Electrochemical bioassay

Creatinine samples were prepared in water or artificial urine. Artificial urine was synthesized according to the protocol developed by Sarigul *et al.*[2] In a typical experiment, 120 µL of creatinine sample was mixed with 120 µL of 2X ferricyanide solution, which contained 500 mM ferricyanide, 500 mM acetate pH 5 and 2 M KCl. The sample was then incubated in a thermoshaker (Thermomixer, Eppendorf) at 750 rpm and 65°C for 25 minutes before being analyzed on the electrochemical devices. The injection volumes were 20 µL for the static devices and 2.5 µL for the flow injection devices. For the pH study, the following buffers were used: citrate pH 3, acetate pH 4 and 5, phosphate pH 6 and 6.8, Tris·HCl pH 7.5, 9 and 10. In flow injection analysis, the running buffer consisted of 0.1% BSA, 0.1% Tween 20, 250 mM acetate pH 5 and 1 M KCl. A negative control was typically injected into the device before the first measurement.

Clinical samples

Clinical urine samples were collected from canine and feline adult specimens at the Veterinary Practice les Champs-Neufs in Sion, Switzerland. Samples were anonymized and conserved at -80 °C for further use. It is worth noting that urinary creatinine is unaffected by freeze-thaw cycles.[3] The creatinine content of the 19 clinical samples was analyzed using a commercial colorimetric kit (Creatinine urinary Colorimetric Assay Kit, Cayman Chemical) following the manufacturer's instructions. A sample dilution factor of 30-fold was used. Alkaline picrate solution was added to the samples, and the absorbance at 492 nm was measured after 10 minutes. Absorbance values were corrected by subtracting absorbance values (492 nm) after 20 minutes incubation with the acid solution. Each sample was measured in

triplicate. For electrochemical flow injection, clinical samples were diluted 3-fold before being mixed with the 2X ferricyanide solution.

Statistical analysis

Unless mentioned otherwise, measurements are performed in triplicate (n=3). In the case of bar and scatter plots, error bars represent the median and 25-75% percentile range. For fitted or measured curves, the mean values are represented by a solid curve and the standard deviation in each direction is indicated by a shaded surface. The detection limit is defined as the intersection of the blank signal + 3 standard deviations and the linear response curve from the lowest detectable concentration.[4] The sensitivity refers to the calibration sensitivity, i.e. the slope of a linear fit to the signal-concentration response.[4] The standard deviation of the calibration sensitivity is a measure of the linearity of the sensor response. Raw data are represented unless stated otherwise. MATLAB (R2022b) was used for all the data processing and statistical analysis.

Supplementary Figures

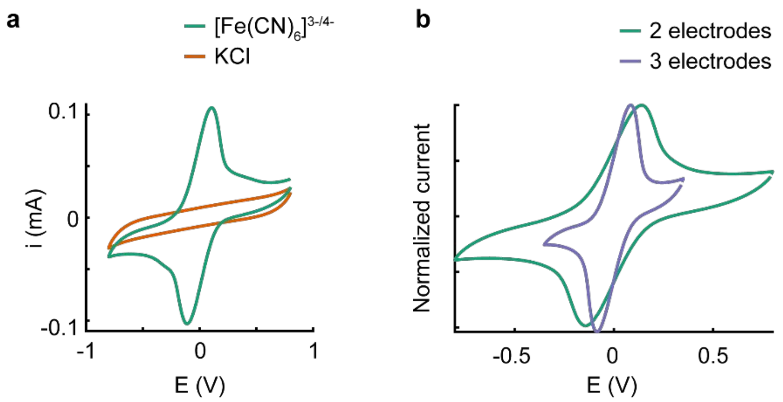

Figure S1: (a) Cyclic voltammogram for 10 mM ferri-/ferrocyanide in 1 M KCl, and 1 M KCl in our two-electrode detector, with a scan rate of 100 mV s^{-1}. (b) Comparison of a two- and three-electrode set-up for detecting 10 mM ferri-/ferrocyanide in 1 M KCl at a scan rate of 80 mV s^{-1}. The three-electrode setup comprises a pseudo-reference electrode (bare carbon). A peak splitting of 169 mV peak splitting is observed for 3 electrodes, and 285 mV for 2 electrodes, indicative of diffusion limitations on either the WE or CE in the case of the reference-free two-electrode configuration.

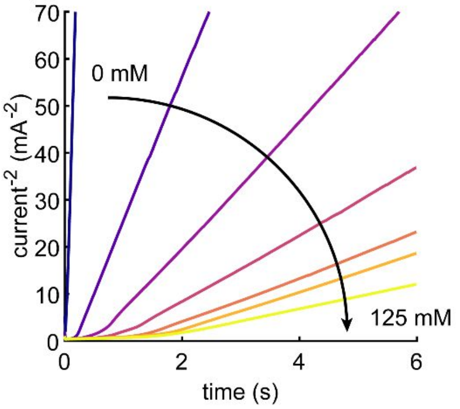

Figure S2: Current decay over time after a step potential of 0.35 V for an increasing ratio of ferrocyanide from 0 mM to 125 mM (total concentration of ferro-/ferricyanide of 250 mM in 1 M KCl). The inversed square currents are plotted according to the Cottrel equation, i.e. $i^{-2} = \mathrm{const} \cdot t$. As the reaction of the CE becomes progressively more limiting the response deviates from the ideal response, with a shift in time before the linear region.

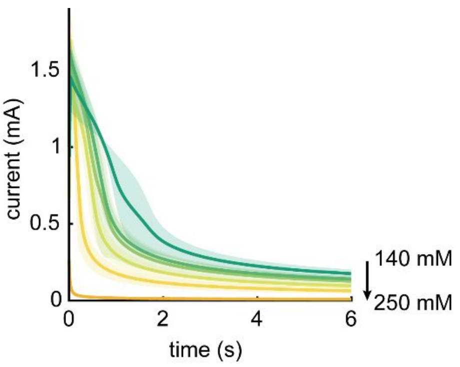

Figure S3: Current decay over time after a step potential of 0.35 V for an increasing ratio of ferrocyanide from 140 mM to 250 mM (total concentration of ferro-/ferricyanide of 250 mM in 1 M KCl). Despite an increasing amount of ferrocyanide available for oxidation, the signal magnitude decreases due to CE limitations (*n*=3).

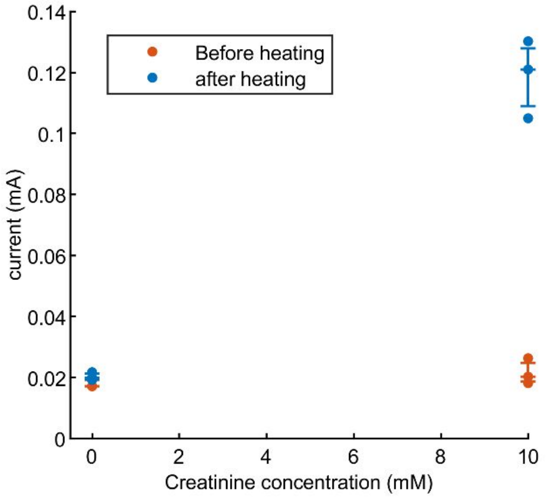

Figure S4: Negative and positive controls for the reaction of creatinine with 250 mM ferricyanide in 250 mM acetate pH 5 and 1 M KCl. Experiments were repeated in triplicate, incubated for 10 minutes at 65°C. No signal increase is observed in the absence of the heating step. Conversely, the heating step does not lead to a change in signal.

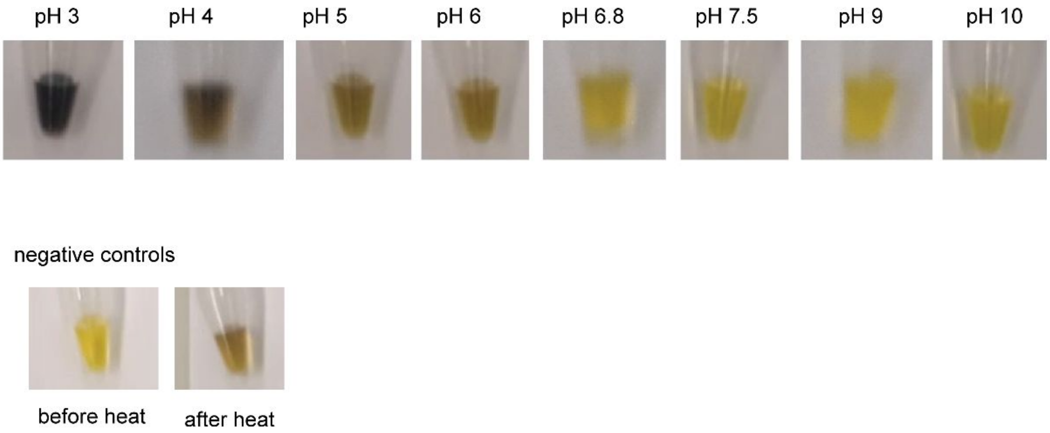

Figure S5: Images of the vials following the incubation of 10 mM creatinine in 250 mM ferricyanide, 1 M KCl and 250 mM buffers with various pH for 10 minutes at 65°C. Under acidic conditions, a deep blue color indicative of the formation of Prussian blue, i.e. $Fe^{III}_4[Fe^{II}(CN)_6]_3$, is observed. This side reaction is undesired as it lowers the reaction efficiency and potentially leads to the evolution of cyanide gas (up to 1 ppm HCN detected just above the liquid surface).

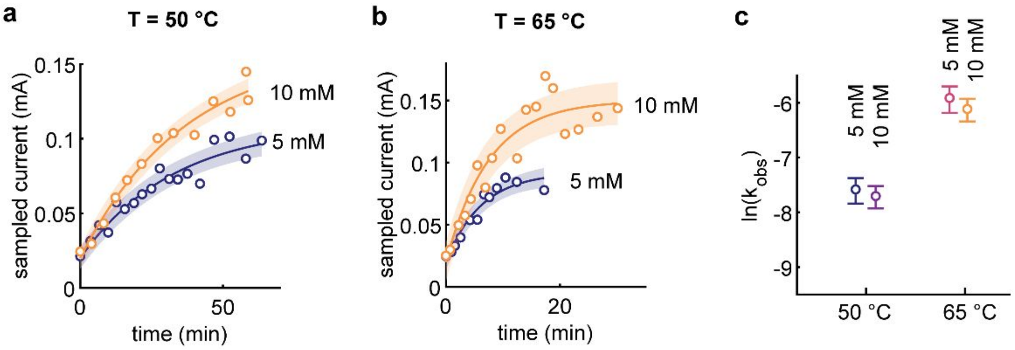

Figure S6: Kinetic measurements of the reaction of creatinine with 250 mM ferricyanide in 250 mM acetate pH 5 and 1 M KCl. (a,b) Reaction kinetics were evaluated and compared for 5 or 10 mM creatinine, and at 50°C or 65°C. (c) The observed kinetic parameter, k_{obs}, was insensitive to creatinine concentration, consistent with our assumption of pseudo first-order kinetics.

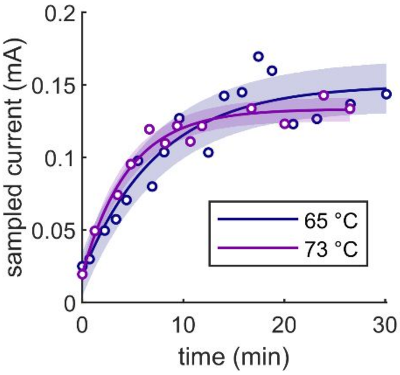

Figure S7: Kinetic measurements for a concentration of 10 mM creatinine at 65°C or 73°C reacting with 250 mM ferricyanide in 250 mM acetate pH 5 and 1 M KCl. Whilst the kinetics followed the Arrhenius principle up to 65°C, further temperature increases do not lead to faster kinetics, indicative of mass transport limitations (controlled by the mixing).

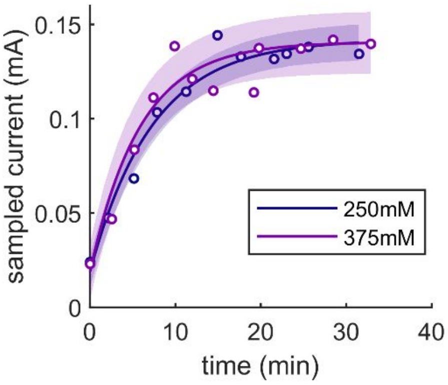

Figure S8: Kinetic measurements for a concentration of 10 mM creatinine with 250 or 375 mM ferricyanide at a temperature of 65°C. While we observe a first-order influence of ferricyanide concentration on the kinetics up to 250 mM, at 375 mM the reaction speed is not improved, indicative of mass transport limitations.

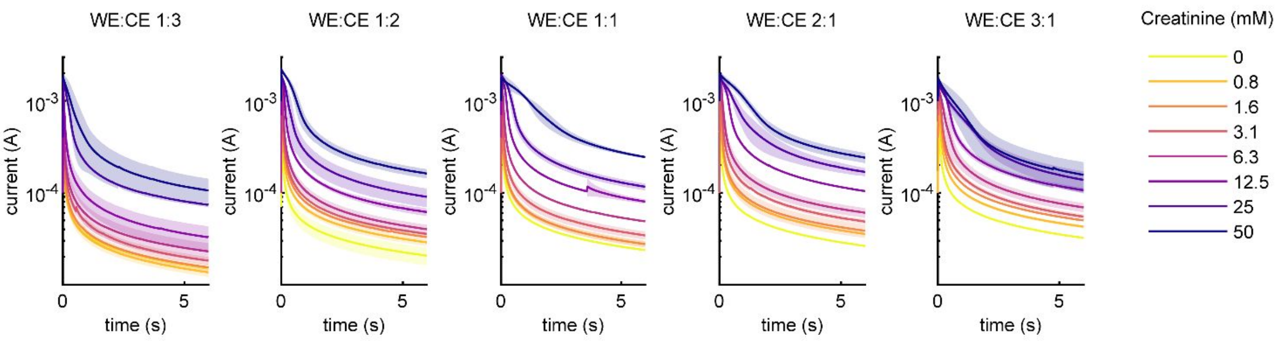

Figure S9: Chronoamperometric measurements of creatinine spiked in artificial urine samples. The samples were mixed 1:1 with a 2X solution of reagents comprising 500 mM ferricyanide, 500 mM acetate pH 5 and 2 M KCl. Data were collected in triplicate on the static sensor designs, and the relative size of the WE compared to the CE was steadily increased (left to right). The response curves were computed from the currents sampled at 5 seconds.

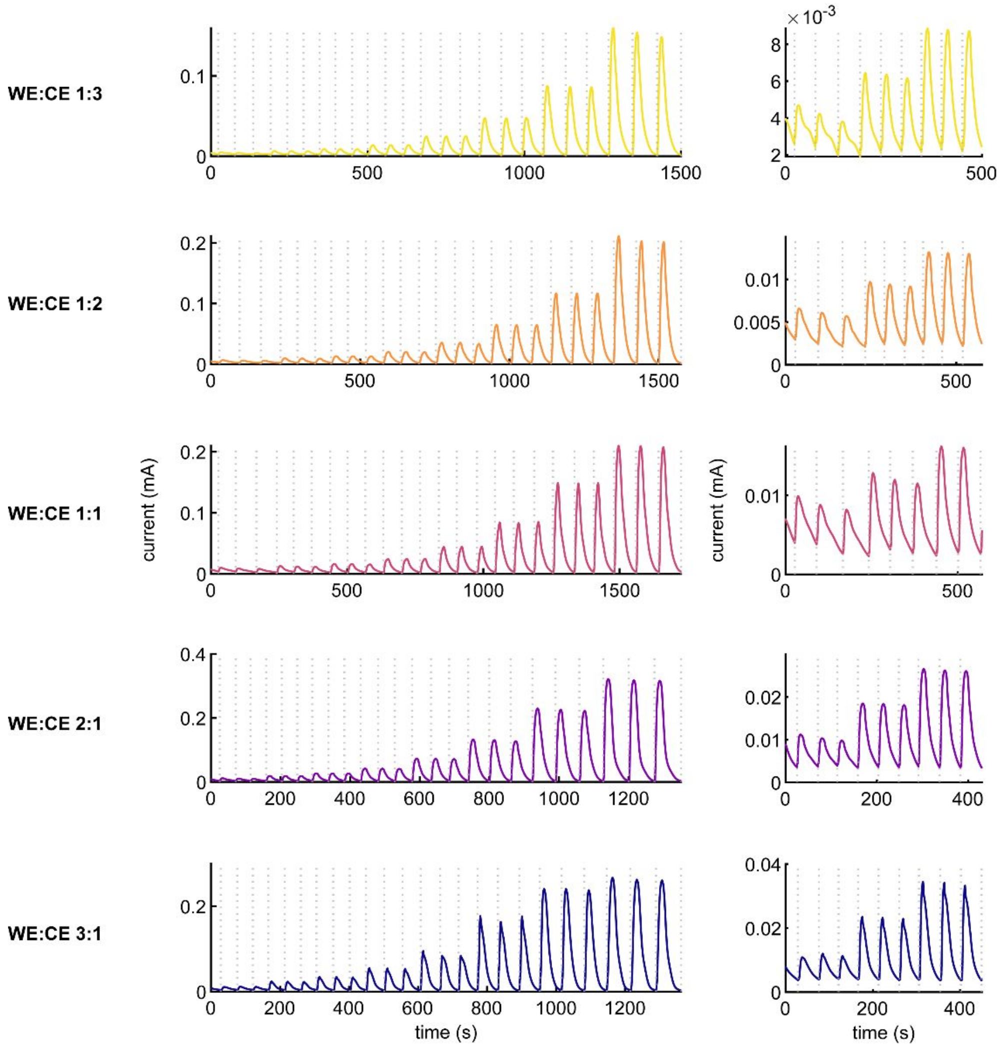

Figure S10: Flow analysis of spiked artificial urine samples with triplicate injections of creatinine concentrations of 0, 0.8, 1.6, 3.1, 6.3, 13, 25 and 50 mM. The relative size of the WE compared to the CE is increased from 1:3 to 3:1. Panels on the right hand side display the first nine injections at the three lowest concentrations, including the negative controls. The non-zero response of the negative controls originates from the presence of trace amounts of ferrocyanide in the ferricyanide solution.

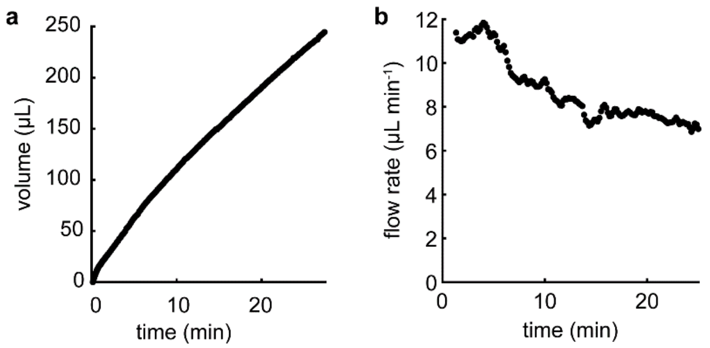

Figure S11: Flow of buffer (250 mM acetate pH 5 and 1 M KCl) through the paper-based microfluidics channel (4 mm×150 µm, width×depth). (a) The volume of buffer absorbed over time was measured gravimetrically and (b) the flow rate was calculated from the numerical derivation of the absorption curve. After the wetting phase, the Darcy flow reached 12 µL min^{-1}, before decreasing over 25 minutes down to 7 µL min^{-1}.

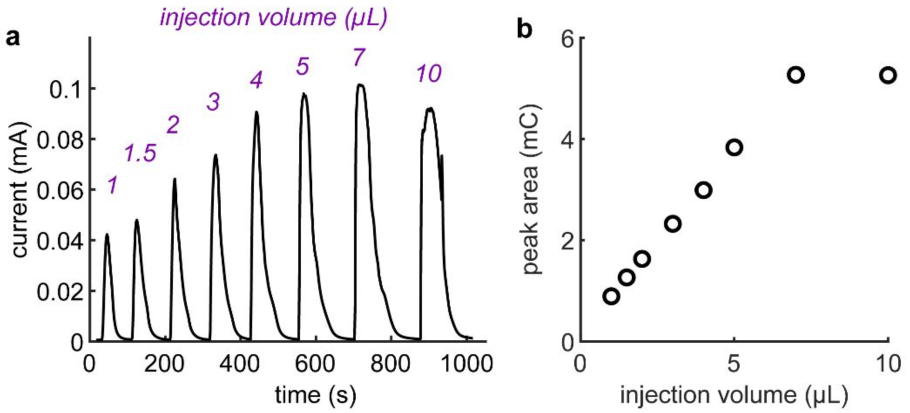

Figure S12: (a) Current response following the injection of a 10 mM spiked creatinine sample with increasing volumes, from 1 to 10 µL. (b) The peak area increased proportionally to the injected volume up to 7 µL, above which the port cavity saturates and floods.

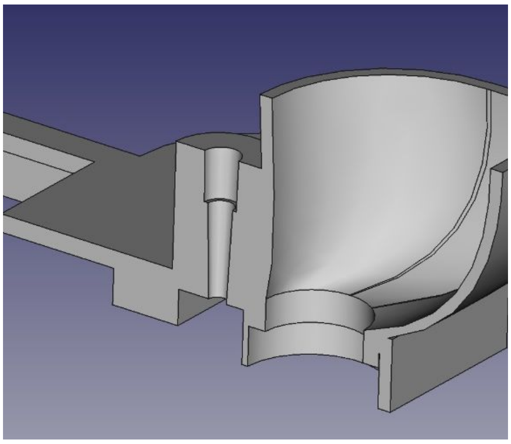

Figure S13: Cross section of the 3D-printed flow injection device housing. The buffer reservoir and flow injection port can be seen on the right and center of the image, respectively. The injection port interfaces tightly with the pipette tip to maximize device reliability and reduce signal variations from injection to injection.

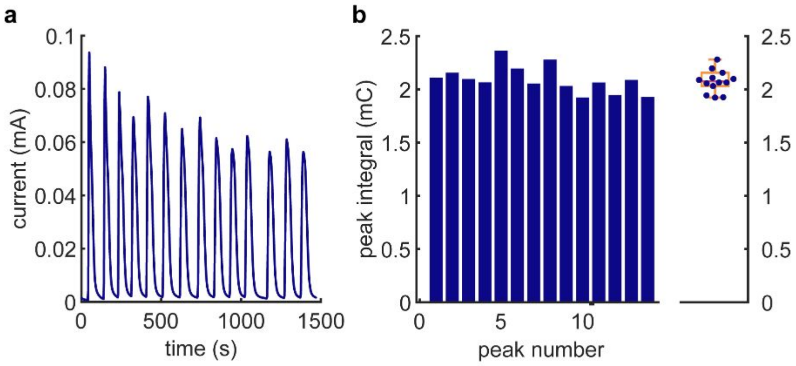

Figure S14: Reproducibility of the signal following multiple sequential injections of 2.5 µL of 10 mM spiked creatinine sample. Whilst the peak height decreases and broadens over time due to the slower flow rate (a), the peak integral remained constant with a 6% standard deviation of the signal over 14 injections (b).

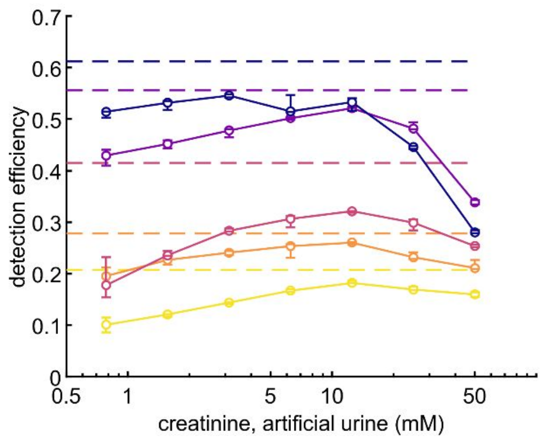

Figure S15: Detection efficiency calculated from the integrated peak signals from Fig. S10. The dashed lines indicate the maximum efficiency that can be theoretically reached considering the partial coverage of the WE in the channel.

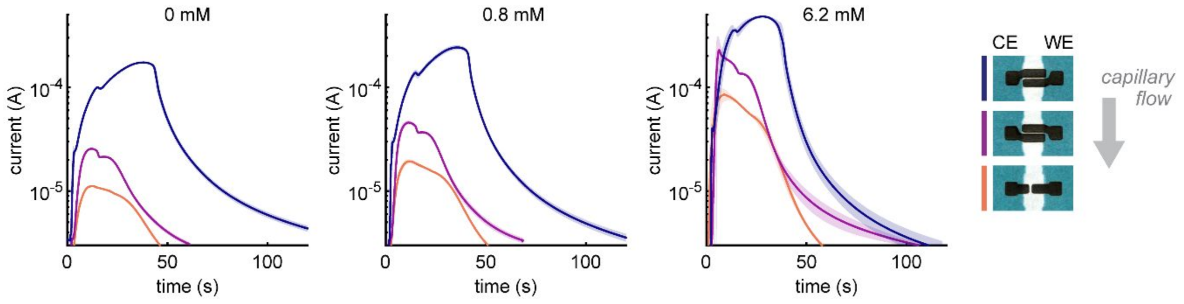

Figure S16: Current response in flow injection analysis following the injection of artificial urine samples with 0, 0.8 or 6.2 mM creatinine with various arrangements of the two electrodes (n=3).

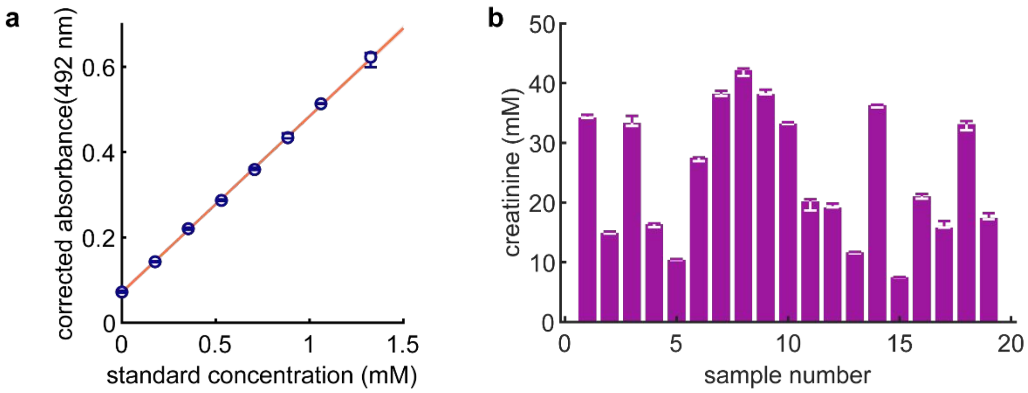

Figure S17: Measurement of creatinine content in clinical urine samples using a commercial colorimetric test from Cayman Chemical. (a) The assay was calibrated using creatinine standards (*n*=3) provided by the manufacturer in a two-step procedure on a plate reader. (b) Clinical samples were measured using the same procedure (*n*=3), and their creatinine content computed using the calibration curve.

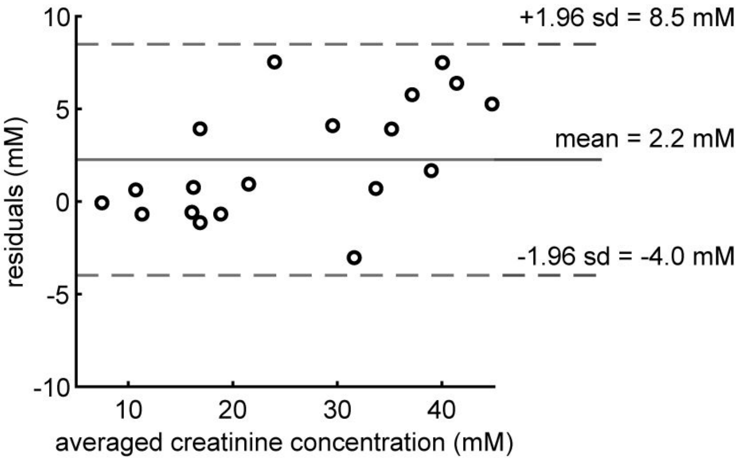

Figure S18: Bland–Altman plot comparing the performance of our FIA device with the commercial colorimetric test on a plate reader. The mean and 95% confidence interval of the residuals across the average creatinine concentration are indicated.

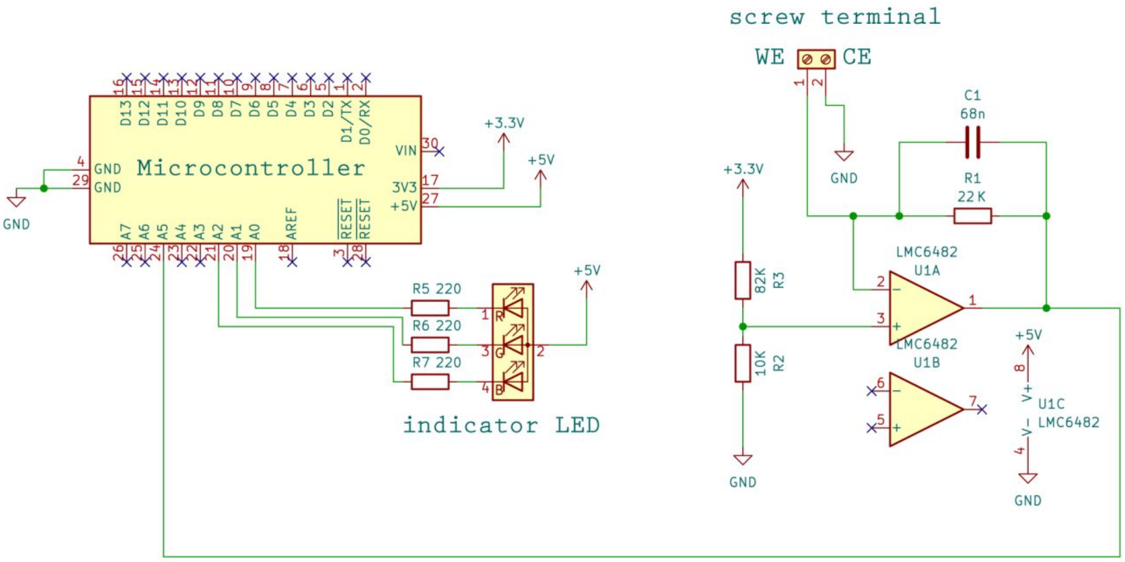

Figure S19: Circuit schematic of the low-cost smart reader, including a microcontroller, an RBG LED indicating the status of the measurement, and an operational amplifier to apply and monitor the current. The current range is set by R1, and R1=22 kΩ corresponds to currents from -16 µA to 211 µA with 0.2 µA resolution.

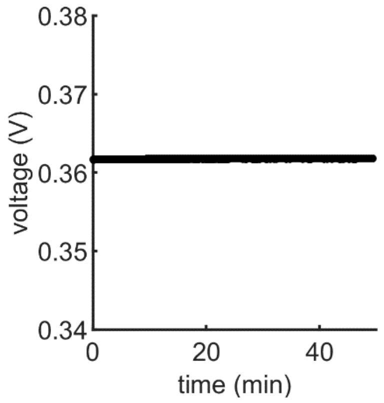

Figure S20: Stability of the applied voltage from the smart reader. The voltage is set by the voltage divider and was measured as 361.76±0.05 mV over 45 minutes.

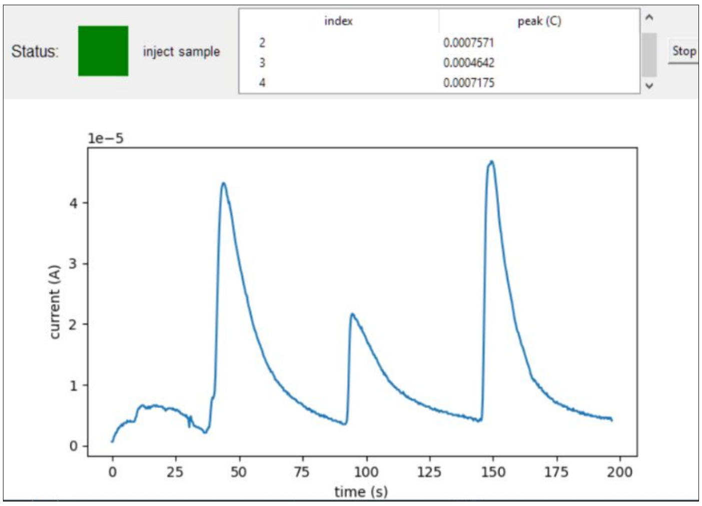

Figure S21: Python graphical user interface for the smart reader, showing the status of the measurement, the peak indices, and integrated signals, as well as the current time response. All computations and analysis are performed on the reader and communicated to the display via USB.

Supplementary Tables

Table S1: Summary of the information on the clinical samples and subsequent creatinine analysis, including a comparison of the flow electrochemical detection benchmarked against the commercial gold-standard assay.

**Sample**			**Creatinine analysis**			
**Nr**	**Collection date**	**Type**	**Commercial colorimetric method**	**Flow electrochemical detection**	**Deviation**	**Relative deviation**
**1**	17.04.2023	feline	34.3 mM (34.2-34.7)	40.0 mM (38.7-41.4)	5.8 mM	0.17
**2**	17.04.2023	canine	14.9 mM (14.8-15.1)	18.8 mM (17.7-19.9)	3.9 mM	0.26
**3**	17.04.2023	feline	33.3 mM (32.8-34.5)	34.0 mM (32.9-35.2)	0.7 mM	0.02
**4**	17.04.2023	canine	16.4 mM (15.9-16.5)	15.8 mM (14.6-17.0)	-0.6 mM	-0.04
**5**	18.04.2023	canine	10.4 mM (10.4-10.5)	11.0 mM (9.7-12.3)	0.6 mM	0.06
**6**	26.04.2023	feline	27.5 mM (26.9-27.5)	31.6 mM (30.5-32.7)	4.1 mM	0.15
**7**	27.04.2023	feline	38.2 mM (37.9-38.7)	44.6 mM (43.1-46.2)	6.4 mM	0.17
**8**	27.04.2023	feline	42.2 mM (41.2-42.4)	47.4 mM (45.7-49.1)	5.3 mM	0.12
**9**	27.04.2023	feline	38.2 mM (38.1-38.9)	39.8 mM (38.5-41.2)	1.7 mM	0.04
**10**	27.04.2023	feline	33.2 mM (33.1-33.5)	37.1 mM (35.9-38.4)	3.9 mM	0.12
**11**	01.05.2023	canine	20.2 mM (18.7-20.6)	27.8 mM (26.7-28.8)	7.5 mM	0.37
**12**	02.05.2023	feline	19.2 mM (19.2-19.8)	18.5 mM (17.4-19.6)	-0.7 mM	-0.04
**13**	02.05.2023	feline	11.7 mM (11.5-11.7)	11.0 mM (9.6-12.3)	-0.7 mM	-0.06
**14**	02.05.2023	canine	36.3 mM (35.9-36.4)	43.8 mM (42.3-45.3)	7.5 mM	0.21
**15**	02.05.2023	feline	7.5 mM (7.5-7.5)	7.4 mM (6.0-8.9)	-0.1 mM	-0.01
**16**	02.05.2023	feline	21.0 mM (20.6-21.4)	22.0 mM (20.9-23.0)	0.9 mM	0.04
**17**	02.05.2023	feline	15.8 mM (15.8-16.9)	16.6 mM (15.5-17.8)	0.8 mM	0.05
**18**	05.05.2023	feline	33.1 mM (32.1-33.7)	30.1 mM (29.0-31.2)	-3.0 mM	-0.09
**19**	05.05.2023	canine	17.4 mM (17.2-18.2)	16.3 mM (15.1-17.5)	-1.1 mM	-0.07

Table S2: Selected interfering substances present in urine. The clinical and abnormal concentrations were taken from Refs [5] and [6]. The last column indicates the selected concentrations of each substance for the interference test.

Substance	Normal range	Abnormal	Interference test
**NaCl**	5 – 20 g/L (75-300 mM)		200 mM (11.7 mg/mL)
**Urea**	10 – 35 g/L (160-350 mM)	> 100:1 urea:Cr	500 mM (30 mg/mL)
**Glucose**	0.1 – 0.8 mM	> 5 mM	10 mM (1.8 mg/mL)
Ketone **(3-hydroxybutyrate)**	<200 mg/L	> 800 mg/L	7.5 mM (0.78 mg/mL)
**Albumin**	10 – 140 mg/L	> 300 mg/L	1 g/L
**Cystine**	< 38 mg/L (0.32 mM)	>75 mg/g Cr (~ 0.45 mM)	0.5 mM (0.12 mg/mL)
**Uric acid**	< 6 mM	> 1 UA:Cr	10 mM (1.7 mg/mL)
**Bilirubin**	negative	> 15 mg/L (25 uM)	25 uM (15 mg/L)
**Ascorbic acid**	negative	> 0.6 mM	1 mM (0.18 mg/mL)

Table S3: Bill of materials for the smart reader. The prices are indicated for a purchase volume of 1000 units.

Item	Model	Unit price (USD)	Link
Microcontroller	Atmega328p	0.90	https://www.aliexpress.com/item/1005002976480289.html
Dual Op-amp	LMC6482IM	0.60	https://www.ti.com/product/LMC6482/part-details/LMC6482IM/NOPB
Resistors (×6)		0.20	
Capacitor		0.35	https://mou.sr/3OKzvi1
RGB LED		0.32	https://mou.sr/3ovF1dU
Screw Terminal		0.40	https://mou.sr/3MZwGbK
	**Total**	**2.77 USD**	

Supplementary Videos

Supplementary Video 1: Illustrative video of a typical experiment, showing the operation of the FIA device on the left, and the corresponding graphical user interface driven by the reader. The device is in idle state until the buffer flow front is detected. Then, a sample is injected, and the associated response monitored and integrated over time until it decays under a current threshold.

References

(1) Bezinge, L.; Lesinski, J. M.; Suea-Ngam, A.; Richards, D. A.; deMello, A. J.; Shih, C. Paper-Based Laser-Pyrolyzed Electrofluidics: An Electrochemical Platform for Capillary-Driven Diagnostic Bioassays. *Adv. Mater.* **2023**, 2302893.

(2) Sarigul, N.; Korkmaz, F.; Kurultak, İ. A New Artificial Urine Protocol to Better Imitate Human Urine. *Sci. Rep.* **2019**, *9* (1), 1–11.

(3) Garde, A. H.; Hansen, Å. M.; Kristiansen, J. Evaluation, Including Effects of Storage and Repeated Freezing and Thawing, of a Method for Measurement of Urinary Creatinine. *Scand. J. Clin. Lab. Invest.* **2009**, *63* (7–8), 521–524.

(4) Long, G. L.; Winefordner, J. D. Limit of Detection A Closer Look at the IUPAC Definition. *Anal. Chem.* **1983**, *55* (07), 712A-724A.

(5) Rifai, N.; Horvath, A. R.; Wittwer, C. .; Tietz, N. W. *Tietz Textbook of Clinical Chemistry and Molecular Diagnostics*; Elsevier, St. Louis, Missouri, 2018.

(6) McPherson, R. A.; Pincus, M. R. *Henry's Clinical Diagnosis and Management by Laboratrory Methods E-Book*; Elsevier Health Sciences, 2021.
